# Neurodegenerative consequences of electrocautery-assisted circumcision on spermatogenesis: A stereological preliminary experimental study in rabbits

**DOI:** 10.1097/MD.0000000000043793

**Published:** 2025-08-08

**Authors:** Binali Firinci, Çetin Aydin, Dilek Yunluel, Ozgur Caglar, Ali Ahiskalioglu, Mehmet Dumlu Aydin

**Affiliations:** a Department of Pediatric Surgery, Medical Faculty of Ataturk University, Erzurum, Turkey; b Department of Pediatric Surgery, Etlik City Hospital, Ankara, Turkey; c Department of Anesthesiology and Reanimation, Medical Faculty of Ataturk University, Erzurum, Turkey; d Department of Neurosurgery, Medical Faculty of Ataturk University, Erzurum, Turkey.

**Keywords:** circumcision, Onuf nucleus, pudendal nerves, sperm

## Abstract

**Background:**

Despite being a widely practiced and debated procedure for centuries, the impact of different circumcision techniques on spermatogenesis remains insufficiently explored. This study investigates the potential effects of conventional surgical and monopolar electrocautery-assisted circumcision methods on spermatogenesis.

**Methods:**

The study was conducted on 18-month-old male New Zealand rabbits, divided into 3 groups: a control group (Group I, n = 5), a surgical circumcision group without electrocautery (Group II, n = 5), and a monopolar electrocautery group (Group III, n = 8), with a total of 18 animals. All procedures were performed under general anesthesia. The animals were monitored for 3 weeks before being sacrificed. Stereological analysis was used to evaluate Onuf nucleus, pudendal ganglia, and sperm counts. Histopathological analyses were performed on the affected tissues to evaluate structural and cellular changes.

**Results:**

Degenerative changes were observed in the dorsal penile fibers of the pudendal nerves, the pudendal ganglia, and Onuf nucleus. The density of degenerative neurons (n/mm³) in Onuf nucleus and pudendal ganglia, along with sperm counts, were as follows: Group I: 4 ± 1, 9 ± 3, and 96,891 ± 11,893, respectively; Group II: 11 ± 4, 154 ± 45, and 93,156 ± 8365; Group III: 121 ± 34, 2121 ± 318, and 76,783 ± 4956. The *P*-values for comparisons were as follows: Group I versus Group II, *P* < .005; Group II versus Group III, *P* < .0005; and Group I versus Group III, *P* < .00001.

**Conclusion:**

The use of monopolar electrocautery during circumcision may be associated with neurodegenerative changes that originate at the distal pudendal nerve endings and extend retrogradely to the pudendal ganglia and Onuf nucleus. These changes may disrupt testicular innervation and could potentially contribute to reduced sperm production.

## 1. Introduction

Circumcision has been performed for over 4000 years for theological, cultural, and social reasons and remains a widely practiced surgical procedure today. While the removal of the prepuce is traditionally regarded as a minor intervention, the use of electrocautery in penile surgery introduces the risk of neurovascular complications.^[1]^ Electrocautery is commonly employed in surgical procedures to achieve dissection and hemostasis,^[2]^ but its application during circumcision can expose surrounding tissues to electrical currents, leading to iatrogenic damage to structures such as the dorsal root ganglia^[1]^ and the urethral meatus.^[3]^ These injuries may contribute to erectile dysfunction, which can have profound implications for male fertility and overall quality of life for both patients and their partners.^[4]^

The autonomic nervous system plays a crucial role in regulating the function of the urinary and reproductive organs. The parasympathetic innervation of the penis originates from the sacral spinal parasympathetic center.^[5,6]^ A study by Aydin et al reported that spinal cord injury disrupts the modulation of sensory structures within the male urethra, impairing orgasmic perception.^[7]^ Similarly, the use of electrocautery in urogenital surgeries has been associated with damage to penile nerves and pelvic ganglia, potentially leading to long-term neurological deficits.^[8]^ Neural structures are capable of transmitting electrical signals in both anterograde and retrograde directions,^[9]^ and retrograde transport plays a critical role in the pathophysiology of electrical injury in autonomic organs.^[10]^ Severe ischemic injury to the dorsal root ganglia can have significant consequences, including adverse effects on intestinal function.^[11]^ Additionally, cavernous nerve injury following abdominopelvic surgeries has been linked to sexual dysfunction.^[12]^ Erectile dysfunction-related infertility remains a major concern in male reproductive health; however, the impact of penile surgery on spermatogenesis remains an underexplored area of research.

Electrocautery has been shown to cause spinal nerve injuries during spinal surgeries, contribute to neurodegeneration in dorsal root ganglia, and induce femoral artery damage as well as thrombotic occlusion in nerve root arteries.^[13–16]^

In the context of penile surgery, electrocautery has also been associated with taste bud atrophy in the urethral meatus, which may contribute to diminished orgasmic sensation. Given these findings, our study aims to investigate the potential effects of electrocautery-induced pudendal nerve injury on testicular innervation and sperm production. Despite significant advancements in medical and surgical techniques, this issue has not been adequately studied. We hypothesized that the use of monopolar electrocautery during circumcision may induce retrograde neurodegeneration in the pudendal nerve and its central connections, leading to impaired testicular innervation and reduced spermatogenesis.

## 2. Materials and methods

The animals used in this study were owned and managed by our institution. Ethical approval for the study was obtained from the Institutional Review Board of Animal Experiments Local Ethics Committee of Ataturk University (September 17, 2018/10). Eighteen New Zealand rabbits were used in the study. The animals were 18 months old and weightiness 3.3 ± 0.6 kg. Each group was placed in stainless cages (3–4 animals per cage) under stable terms, inclusive of environmental conditions (12-h light/dark cycles, temperature 23 ± 2 °C, light intensity 350–400 lux, humidity 30–60%) was housed within and freedom of water and nutrition. Animals were kept under controlled room conditions. Animals were randomly allocated into 3 groups (Group I: control, n = 5; Group II: surgical circumcision without electrocautery, n = 5; Group III: circumcision with monopolar electrocautery, n = 8) using the RAND function in Microsoft Excel to generate random numbers, ensuring unbiased group assignment. Allocation was concealed by having an independent researcher who was not involved in the intervention or evaluation stages assign and code the animals. Animals were followed in standard conditions cages for 3 weeks after the intervention. Anesthesia was performed by subcutaneous injection of a mixture of ketamine hydrochloride (25 mg/kg), lidocaine hydrochloride (15 mg/kg), and acepromazine (1 mg/kg). Penis disinfection was performed with povidone-iodine. An anteroposterior midline incision was made in the prepuce. A urethral catheter was placed into the animals’ urethra. This practice was performed without the use of electrocautery in Group II and using cautery in Group III. As in the study of Günal et al,^[17]^ cautery energy level of 15 W was preferred in an area of almost 2 mm on the ventral and dorsal side of the tip of the penile body, lasting 5 seconds.^[17]^ After the incision, the prepuce was cauterized. The incision was closed with continuous suture technique using polyglactin suture (7/0). Antibacterial and painkillers were used after the procedure. After surgical site sterilization, prepuce slit was made with surgical clippers and monopolar cautery (20 W/400 kHz, Petas-Petkot 600 [Petas, Ankara, Turkey]). At the end of experimental duration, all animals were then take/end the life of under general anesthesia, the gluteus maximus and medius muscles were separated and the pudendal nerves were exposed. A laminectomy was performed from the sacral 1 (S1) level to the sacral 4 (S4) level, and the pudendal ganglia were removed at the sacral 4 (S4) level of the spinal cord. Additionally, the edges of the ventral urethra were dissected from the tunica and removed together with the neurovascular partition. All tissue samples were anonymized and labeled with numeric codes. Blinding was rigorously applied during histological and stereological evaluations. These analyses were performed by a single experienced pathologist who was blinded to group allocations. The pathologist received only the coded samples and had no access to any information regarding group identities during microscopic evaluation. All removed tissues were fixed in 10% formalin solution for 1 week and then embedded in paraffin blocks. Blocks divided into 5-micrometer sections and stained with hematoxylin and eosin. The preparations were viewed under a microscope at 4× and 40× magnification. Pathological analyses were done using a light microscope (Nikon Co., Tokyo, Japan) by a pathologist. To calculating the density of neurons in pudendal ganglia^[17]^ and sperm numbers, the stereological method used in the study of Çağlar et al was used.^[18]^ Specimens stained with HE, GFAP, and TUNEL methods. Statistical analysis was undertaken using the one-way ANOVA test in SPSS 20.0 for Windows (SPSS Inc., Chicago, IL) (A *P*-value of < .05 was considered statistically significant).

## 3. Results

### 3.1. Anatomical findings

All animals survived except one that was lost during surgery. No somato-sensory or autonomic dysfunction was observed during follow-up. Macroscopically, wound healing was generally uneventful, with the exception of 1 case of phimosis in Group III. Microscopic evaluation revealed varying degrees of edema, vascular engorgement, and neuromatous structures in the penile and perineural tissues across all groups. However, no statistical comparisons were performed between groups for these findings.

### 3.2. Histopathological results

**Figure 1.**
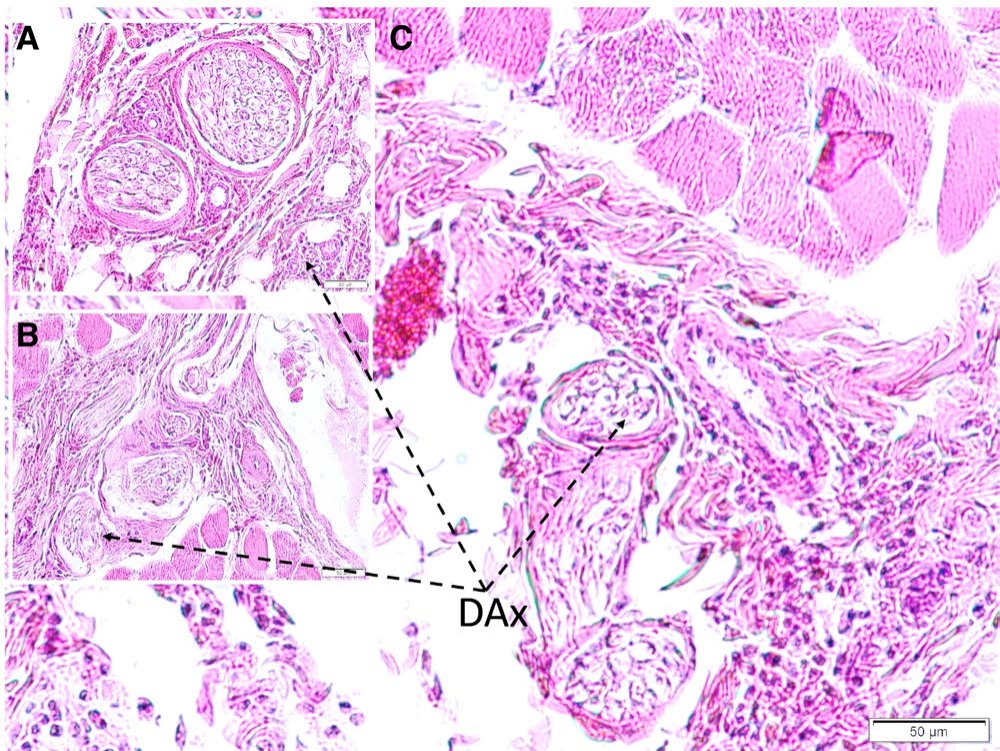
Histopathological appearance of pudendal nerves in Group I. Few degenerated axons (DAx) observed due to aging and tissue preparation latency (A). The more degenerated axons (DA) are seen in Group II (B). Interestingly, the most degenerated axons (DA) are seen in Group III (C) (LM, H&E, 20×).

**Figure 2.**
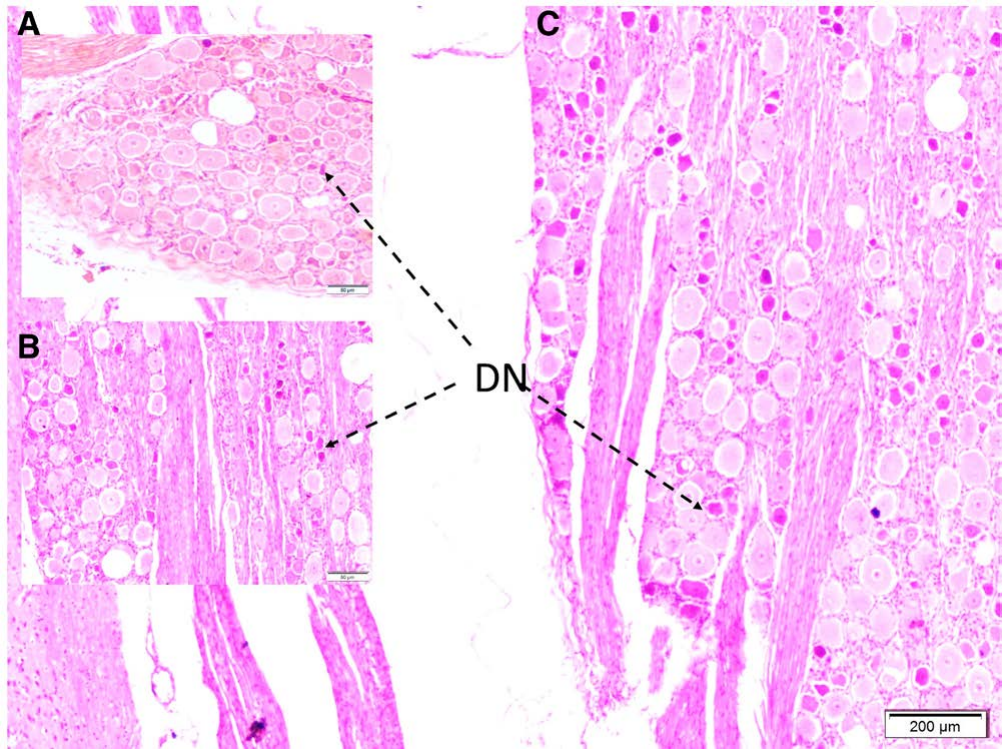
Histopathological appearance of dorsal root ganglion in Group I. Few degenerated neurons (DN) observed due to aging and tissue preparation latency (A). The more degenerated neurons (DN) are seen in Group II (B). Interestingly, the most degenerated neurons (DN) are seen in Group III (C) (LM, H&E, 4×).

**Figure 3.**
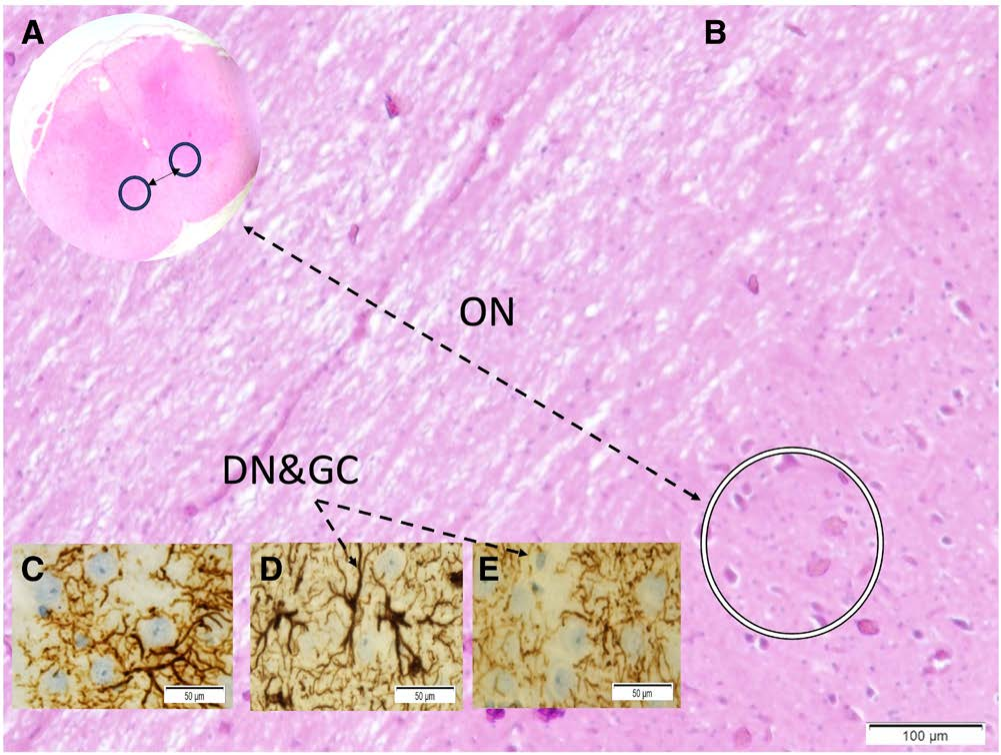
Onuf nucleus (ON) in anteriorly located in gray matter (A), darkened degenerated neurons (DN) (B) and deformed neurons together with glial cells in Group I (C), Group II (D) and Group III are observed (LM, H&E/A,B; and GFAP/C-E; 10×).

**Figure 4.**
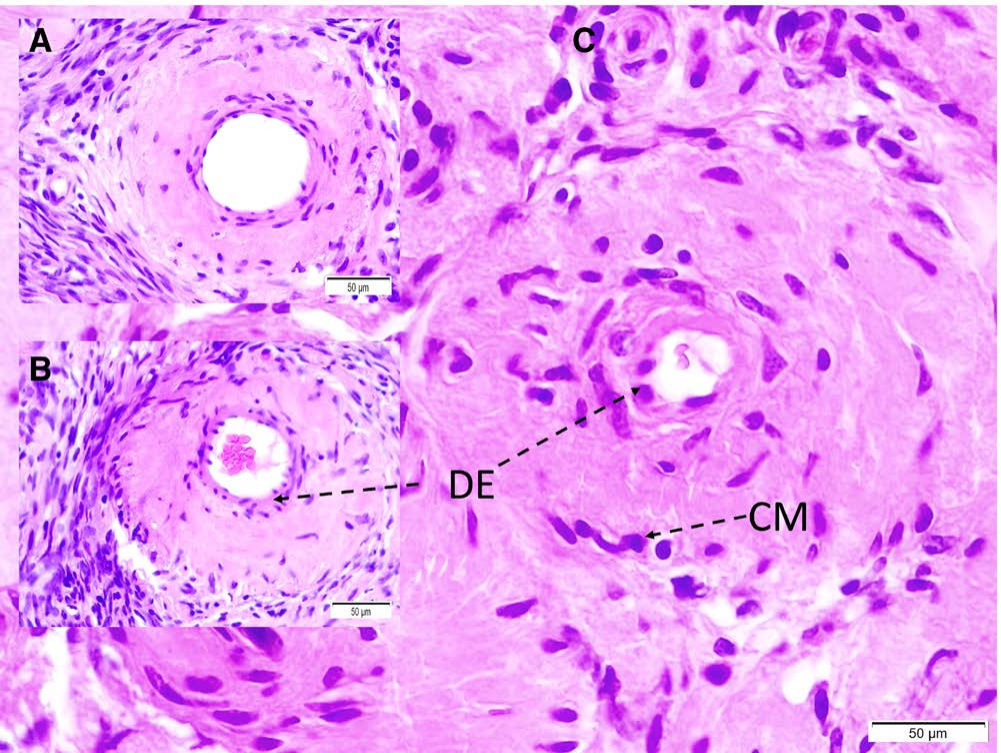
Testicular arteries in a Group I (A), Group II (B), and Group III (C). It is noticed that testicular arteries is moderately constructed with minimally degenerated endothelial cells and narrowed lumen in Group II (B) and severely constructed and injured desquamated endothelial cells, contracted muscles and importantly narrowed lumen in Group III (C) (LM; H&E, 20×).

**Figure 5.**
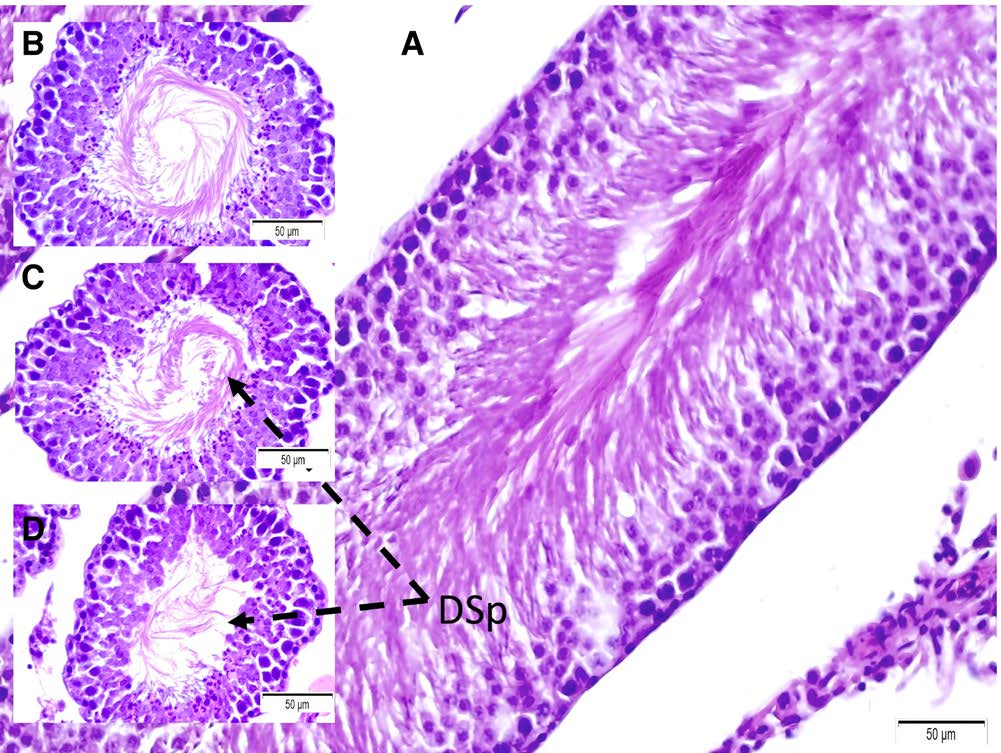
Histopathological appearance of testicular tissues in Group I. Few degenerated sperms (DSp) observed due to aging and tissue preparation latency (A). The more degenerated sperms (DSp) are seen in Group II (B). Interestingly, the most degenerated sperms (DSp) are seen in Group III (C) (LM, H&E, 20×).

**Figure 6.**
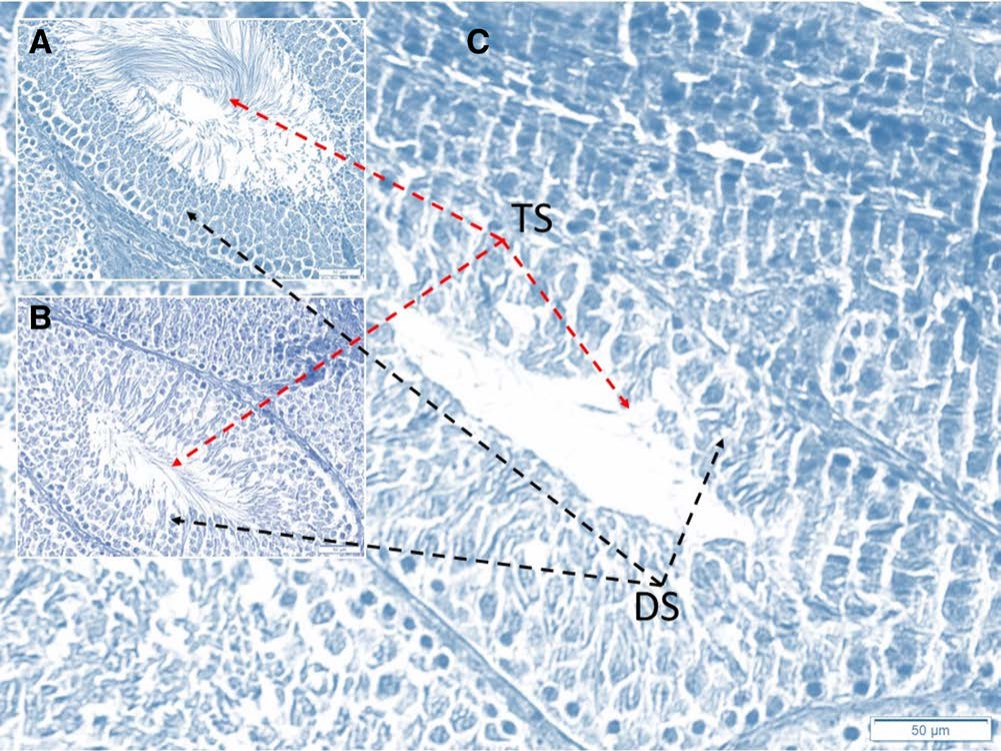
Histopathological appearance of testicular tissues in a animal. Few degenerated-apoptotic sperms (DS) observed due to aging and tissue preparation latency (A). The more degenerated-apoptotic sperms (DS) are seen in Group II (B). Interestingly, the most degenerated-apoptotic sperms (DS) are seen in animals that with monopolar cautery using animals (Group III) (C). At the mean time, the tails of sperms (TS) are more destructed in Group III than in Group II; and Group II more injured than Group I (LM, TUNEL, 20×).

Microscopic evaluation showed fibrous and neurovascular tissue damage, obstructed microvessels in the glandular and distal urethral regions, and prominent vascular congestion. Demyelination and axonal injury were observed in the pudendal nerves (Fig. 1). Degenerated neurons were detected in the pudendal nerve, dorsal root ganglia, and Onuf nucleus (Figs. 2 and 3). Vascular spasm, endothelial injury, and congestion were present in the testicular arteries (Fig. 4). Degenerative changes in spermatogenic cells included nuclear shrinkage, apoptotic features, disrupted sperm tails, and reduced sperm density (Figs. 5 and 6).

### 3.3. Numerical results

Degenerated neuron intensity of the Onuf nucleus (n/mm^3^), the pudendal ganglia (S2) (n/mm^3^) and mean sperm count (n/mm^3^) were calculated as 4 ± 1, 9 ± 3, and 96.891 ± 11.893; in the Group I, 11 ± 4, 154 ± 45, and 93.156 ± 8.365 in the Group II; and 121 ± 34, 2121 ± 318, and 76.783 ± 4.956 in the Group III. *P*-values were *P* < .005 in Group I/Group II; *P* < .0005 in Group II/Group III and *P* < .00001 in Group I/Group III (Table 1).

**Table 1:** Numerical results of degenerated neuron densities of Onuf nucleus, pudendal ganglia, and sperm count.

	GI	GII	GIII
Degenerated neuron densities of Onuf nucleus (n/mm^3^)	4 ± 1†	11 ± 4‡	121 ± 34*
Pudendal ganglia (n/mm^3^)	9 ± 3†	154 ± 45‡	121 ± 318*
Mean sperm count (n/mm^3^)	96.891 ± 8.365†	93.156 ± 8.365‡	76.783 ± 4.956*

GI = group control, GII = group sham, surgical circumcision, GIII = group study, electrocautery.

* *P* < .00001 GI vs GIII, one-way Anova.

† *P* < .005 GI versus GII, one-way Anova.

‡ *P* < .0005 GII versus GIII, one-way Anova.

## 4. Discussion

This study has showed that electrocautery-induced retrograde degeneration of the pudendal nerves may play a crucial role in testicular denervation and sperm production impairment.

The pudendal nerve originates from the sacral spinal cord (S2–S4) and plays a critical role in male sexual function, including erection and ejaculation.^[19,20]^ The Onuf nucleus, first described by Onufrowicz in 1899, is a key component of the sacral parasympathetic system, contributing to the innervation of the genital organs and regulating urinary and sexual functions.^[21]^ Damage to this nucleus, such as in spinal subarachnoid hemorrhage (SAH), has been associated with urinary retention and spermatogenesis impairment.^[18]^ Similarly, circumcision (especially when performed with electrocautery) may induce retrograde degeneration of the pudendal nerves and the Onuf nucleus, leading to testicular denervation and subsequent disruption of sperm production.

The use of monopolar electrocautery carries the risk of uncontrolled thermal diffusion to adjacent neurovascular structures, which may potentiate local ischemia, oxidative stress, or secondary inflammatory cascade factors that could independently contribute to neurodegeneration or spermatogenic impairment. To minimize variability, we standardized the duration and power setting of electrocautery use, and all procedures were performed by the same experienced surgical team.

Electrocautery is commonly employed in penile surgeries but has been associated with significant neurovascular complications. During circumcision, the electrical energy delivered can penetrate deep tissues, potentially damaging the dorsal nerves of the penis and pudendal ganglia. The retrograde transmission of electrical energy may disrupt the neural circuits essential for spermatogenesis, further exacerbating neuronal degeneration in adjacent structures. This mechanism aligns with previous findings demonstrating that electrocautery can induce neurodegeneration in dorsal root ganglia and cause spinal nerve injury.^[13,14]^ Our study supports the hypothesis that electrocautery-induced pudendal nerve damage disrupts the neural regulation of testicular function, potentially leading to reduced sperm count and infertility.

While the detrimental effects of spinal cord injuries and SAH on spermatogenesis are well documented, the specific impact of circumcision and electrocautery on pudendal nerve–Onuf nucleus circuits remains inadequately explored. Previous studies have primarily focused on the acute complications of circumcision, such as infection and bleeding, while largely overlooking its long-term neurovascular consequences. Our findings suggest that circumcision, particularly when performed using electrocautery, may induce retrograde neurodegeneration, leading to testicular denervation and impaired sperm production. This mechanism is consistent with the known effects of spinal cord injuries on testicular function and introduces a novel pathway by which circumcision may contribute to male infertility.

The disruption of the neural plexus between the pudendal nerve and the testes observed in our study underscores the crucial role of the sacral parasympathetic system in regulating spermatogenesis. Degeneration of the pudendal ganglia and Onuf nucleus circuits may lead to testicular denervation, impaired thermoregulation, and subsequent deterioration in sperm quality.^[22]^ These findings align with previous studies demonstrating that spinal cord injuries and SAH can result in decreased sperm count and testicular dysfunction.^[18]^ However, our study is the first to suggest that circumcision performed with electrocautery may similarly lead to neurovascular complications contributing to male infertility.

Although this study was conducted in an animal model, the implications may be meaningful for human clinical practice. Electrocautery is widely used in pediatric and adult penile surgery due to its efficiency in achieving hemostasis. However, our findings raise concerns about potential retrograde neurodegenerative effects following electrocautery-assisted penile surgery. The observed damage to pudendal nerve structures and associated testicular denervation may help explain some cases of idiopathic male infertility or sexual dysfunction that emerge following circumcision, especially in procedures performed with thermal devices. Given the parallels between the autonomic innervation of the reproductive tract in rabbits and humans, these findings suggest that caution is warranted when using electrocautery in penile surgeries.

This study has several limitations. First, the small sample size may restrict the generalizability of our findings. Second, the use of animal models may not fully replicate human anatomical and physiological conditions. The use of 18-month-old rabbits does not perfectly parallel the age at which circumcision is typically performed in human infants. However, our selection was based on anatomical and technical feasibility. Rabbits younger than 3 to 4 months have smaller and more fragile penile structures, which makes standardized circumcision procedures, catheterization, and dissection technically challenging. In contrast, 18-month-old rabbits provide sufficient tissue volume and maturity for safe surgical manipulation and reliable histopathological assessment. Third, the lack of biochemical and radiological data limits our ability to elucidate the underlying mechanisms in greater detail. Future studies should address these limitations by incorporating larger sample sizes, human models, and advanced imaging techniques to validate our findings. Additionally, long-term follow-up data are lacking in our current study. The 3-week study duration was selected to allow sufficient time for the development of retrograde neurodegenerative changes while minimizing the confounding effects of chronic tissue remodeling or spontaneous recovery; however, the transiency or permanency of the observed effects at this time point remains unknown and warrants further investigation. In clinical settings, this could translate into long-term monitoring of reproductive and sexual function in males undergoing electrocautery-assisted penile surgery. Furthermore, as this is an observational experimental study, the results demonstrate associations rather than definitive causal relationships, which should be confirmed in future studies using more robust, longitudinal, and mechanistic designs. Finally, another limitation lies in the limited biophysical characterization of electrocautery application in small animal models. Although power settings and application time were standardized, detailed information regarding current density, thermal diffusion, and tissue conductivity in rabbits was not fully addressed.

## 5. Conclusion

In summary, this study highlights the potential neurovascular complications of circumcision, particularly when performed using electrocautery. The retrograde degeneration of the pudendal nerves and Onuf nucleus circuits may lead to testicular denervation, impaired spermatogenesis, and male infertility. These findings underscore the need for further research into the long-term consequences of circumcision and the development of safer surgical techniques to minimize neurovascular injury.

## Author contributions

**Conceptualization:** Binali Firinci, Ozgur Caglar, Ali Ahiskalioglu, Mehmet Dumlu Aydin.

**Data curation:** Binali Firinci, Çetin Aydin, Dilek Yunluel, Ozgur Caglar.

**Formal analysis:** Çetin Aydin, Dilek Yunluel, Ozgur Caglar.

**Investigation:** Binali Firinci, Çetin Aydin, Dilek Yunluel.

**Methodology:** Binali Firinci, Çetin Aydin, Dilek Yunluel, Ozgur Caglar.

**Software:** Binali Firinci, Mehmet Dumlu Aydin.

**Supervision:** Binali Firinci, Ali Ahiskalioglu, Mehmet Dumlu Aydin.

**Validation:** Ali Ahiskalioglu, Mehmet Dumlu Aydin.

**Visualization:** Binali Firinci, Ali Ahiskalioglu, Mehmet Dumlu Aydin.

**Writing – original draft:** Binali Firinci, Ozgur Caglar, Ali Ahiskalioglu, Mehmet Dumlu Aydin.

**Writing – review & editing:** Binali Firinci, Ali Ahiskalioglu, Mehmet Dumlu Aydin.
